# Tenacious Researchers Identify a Weakness in All Ebolaviruses

**DOI:** 10.1128/mBio.02249-18

**Published:** 2018-11-20

**Authors:** Rebecca M. DuBois

**Affiliations:** a Department of Biomolecular Engineering, University of California Santa Cruz, Santa Cruz, California, USA

**Keywords:** ebola virus, neutralizing antibodies, protein structure-function

## Abstract

The *Ebolavirus* genus has at least five members, four of which are known to cause deadly disease in humans. An ideal therapy or a vaccine would protect against all ebolaviruses, but identifying a common weakness in all of them has remained elusive. West et al. [B. R. West, C. L. Moyer, L. B. King, M. L. Fusco, et al., mBio 9(5):e01674-18, 2018, https://doi.org/10.1128/mBio.01674-18] make the exciting discovery of an “Achilles’ heel,” a cryptic and conserved pocket, on the surface antigen glycoprotein (GP) that is nearly identical in all known ebolaviruses. Key to this discovery was their study of antibody ADI-15878, the only isolated human antibody that can block infectivity of all known ebolaviruses. Following tenacious efforts in X-ray crystallography, West et al. report the high-resolution crystal structures of the Ebola virus GP and the Bundibugyo virus GP, each bound to antibody ADI-15878. These structures reveal a highly conserved but partially obscured site on the virus GP, providing a foundation for design of vaccine antigens or antiviral therapies.

## Abstract (precis)

The *Ebolavirus* genus has at least five members, four of which are known to cause deadly disease in humans. An ideal therapy or a vaccine would protect against all ebolaviruses, but identifying a common weakness in all of them has remained elusive.

## COMMENTARY

The 2013-2016 Ebola virus pandemic, which infected 28,000 people and had 41% lethality, was a stark reminder that for any virus with pandemic potential, vaccines and antiviral therapies need to be developed and ready for immediate deployment. Since this pandemic, three other Ebola virus outbreaks have occurred, and there have also been several outbreaks of the related Bundibugyo virus and Sudan virus, all with similar symptoms and lethality. While all of these viruses are genetically related and belong to the *Ebolavirus* genus, their surface antigens, called glycoprotein (GP), differ in amino acid sequence by at least 50%, making it difficult to develop a vaccine or treatment that would protect against all of them.

Identifying the common weakness in all ebolaviruses was not obvious, but researchers at The Scripps Research Institute knew that such a weakness existed; they knew this because a human monoclonal antibody called ADI-15878, isolated from a survivor of the 2013-2016 Ebola virus pandemic, had already been shown to “neutralize” or block infectivity of all known members of the *Ebolavirus* genus (1). Where exactly this antibody binds to the surface antigen GP and how it neutralizes ebolaviruses was what the Scripps researchers set out to discover.

**FIG 1:**
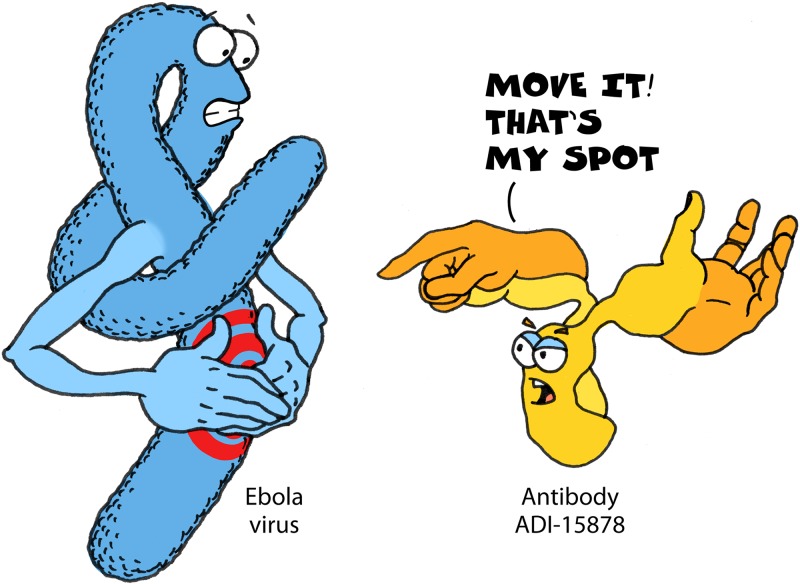
The ADI-15878 antibody binds to a conserved hydrophobic site on the GP glycoproteins that are located on the surface of Ebola virus. This site is normally obscured by a flexible region of GP, represented as the hands of the Ebola virus.

The article by West al. (2) describes the crystal structures of antibody ADI-15878 in complexes with both the Ebola virus GP and the Bundibugyo virus GP. These crystal structures reveal a detailed, atomic view of where ADI-15878 binds to GP. Remarkably, this antibody nudges a flexible region of GP out of the way in order to bind to a greasy (hydrophobic) site underneath it that is highly conserved across the ebolaviruses (Fig. 1). In comparison, most other antibodies studied thus far have been found to stick to the flexible part itself, which is not conserved among different ebolaviruses. Thus, this discovery is a major breakthrough in the field that reveals for the first time a major weakness on the surfaces of all ebolaviruses.

The implications for this study are that researchers can now develop vaccine antigens and antiviral therapies that target this site. In terms of a vaccine, researchers can now pursue the design of vaccine antigens that better expose this important site of vulnerability on GP and elicit broadly neutralizing antibodies, like ADI-15878, that neutralize all ebolaviruses. Hope is bolstered by their other paper, which describes the structure of a mouse antibody that binds to the same site as ADI-15878 but was elicited by repeated vaccination (3). People at the sites of new outbreaks, however, will not have been vaccinated. Importantly, the human antibody ADI-15878 itself may work as a postexposure treatment or a preexposure prophylaxis against all ebolaviruses, which could serve as a strong defense for both patients and medical workers during an emerging epidemic.

What is not immediately obvious from the West et al. article (2) is how technically challenging it was to determine these molecular structures. Tucked into a little box in Table S1 of the West et al. article is the number 10. This number represents the number of submillimeter-sized protein crystals that had to be formed, scooped up, frozen, and then blasted with X-rays in order to acquire enough X-ray diffraction data to make this discovery. Anything less than those 10 crystals was not enough to provide the necessary data, and getting to those best 10 crystals meant years of screening through a myriad of GP constructs, hundreds of crystallization conditions, and hundreds of mediocre crystals to find any that would scatter X-rays well. Achieving any crystals at all with the flexible, heavily glycosylated GP glycoprotein in complex with projecting Fab fragments with additional mobility at the elbow regions required years. While some researchers might have given up during this process, thankfully West and coworkers persevered.

In summary, the article by West et al. (2) is a clear example of how basic scientific breakthroughs are providing a foundation for the development of new therapies that will make our world a better and safer place. In addition, this study reminds us of the human element behind these scientific breakthroughs, driven by researchers that have the determination, perseverance, and tenacity to keep going even when the science gets tough.
